# Supplementary material for: HIVprotI: an integrated web based platform for prediction and design of HIV proteins inhibitors
Source: J Cheminform. 2018 Mar 9;10:12. doi: 10.1186/s13321-018-0266-y (PMC5845081; doi:10.1186/s13321-018-0266-y)
Supplement: Supplementary file 2 — Additional file 2. Source code of HIVProtI web server. [file 13321_2018_266_MOESM2_ESM.zip › HIVprotI_Source-code/hivprotI/weka-3-6-6/doc/weka/experiment/ResultMatrix.html]

ResultMatrix


---


|  |  |  |
| --- | --- | --- |
| **Overview** \| **Package** \| **Class** \| **Tree** \| **Deprecated** \| **Index** \| **Help** \| **Weka's home** | |  |
| **PREV CLASS**   **NEXT CLASS** | **FRAMES**    **NO FRAMES**     **All Classes** |  |
| SUMMARY: NESTED \| FIELD \| CONSTR \| METHOD | DETAIL: FIELD \| CONSTR \| METHOD |  |


---


## weka.experiment Class ResultMatrix

```
java.lang.Object
  weka.experiment.ResultMatrix
```

**All Implemented Interfaces:**: java.io.Serializable, RevisionHandler

**Direct Known Subclasses:**: ResultMatrixCSV, ResultMatrixGnuPlot, ResultMatrixHTML, ResultMatrixLatex, ResultMatrixPlainText, ResultMatrixSignificance

---

``` public abstract class ResultMatrix extends java.lang.Object implements java.io.Serializable, RevisionHandler ```

This matrix is a container for the datasets and classifier setups and
their statistics. Derived classes output the data in different formats.
Derived classes need to implement the following methods:

- `toStringMatrix()`
- `toStringKey()`
- `toStringHeader()`
- `toStringSummary()`
- `toStringRanking()`

**Version:**
:   $Revision: 1.9 $

**Author:**
:   FracPete (fracpete at waikato dot ac dot nz)

**See Also:**: `toStringMatrix()`, `toStringKey()`, `toStringHeader()`, `toStringSummary()`, `toStringRanking()`, Serialized Form

---

| **Field Summary** | |
| --- | --- |
| `java.lang.String` | `LEFT_PARENTHESES`             the left parentheses for enumerating cols/rows |
| `java.lang.String` | `LOSS_STRING`             loss string |
| `java.lang.String` | `RIGHT_PARENTHESES`             the right parentheses for enumerating cols/rows |
| `static int` | `SIGNIFICANCE_LOSS`             loss |
| `static int` | `SIGNIFICANCE_TIE`             tie |
| `static int` | `SIGNIFICANCE_WIN`             win |
| `java.lang.String` | `TIE_STRING`             tie string |
| `java.lang.String` | `WIN_STRING`             win string |


**Constructor Summary**

`ResultMatrix()`
  
          initializes the matrix as 1x1 matrix

`ResultMatrix(int cols,
int rows)`
  
          initializes the matrix with the given dimensions

`ResultMatrix(ResultMatrix matrix)`
  
          initializes the matrix with the values from the given matrix


| **Method Summary** | |
| --- | --- |
| `void` | `addHeader(java.lang.String key, java.lang.String value)`             adds the key-value pair to the header |
| `void` | `assign(ResultMatrix matrix)`             acquires the data from the given matrix |
| `void` | `clear()`             removes the stored data and the ordering, but retains the dimensions of the matrix |
| `void` | `clearHeader()`             removes all the header information |
| `void` | `clearRanking()`             clears the currently stored ranking data |
| `void` | `clearSummary()`             clears the current summary data |
| `double` | `getAverage(int col)`             returns the average of the mean at the given position, if the position is valid, otherwise 0 |
| `int` | `getColCount()`             returns the number of columns |
| `boolean` | `getColHidden(int index)`             returns the hidden status of the column, if the index is valid, otherwise false |
| `java.lang.String` | `getColName(int index)`             returns the name of the row, if the index is valid, otherwise null. |
| `int` | `getColNameWidth()`             returns the current width for the column names |
| `int[]` | `getColOrder()`             returns the current order of the columns, null means the default order |
| `double` | `getCount(int index)`             returns the count for the row. |
| `int` | `getCountWidth()`             returns the current width for the counts |
| `int` | `getDisplayCol(int index)`             returns the displayed index of the given col, depending on the order of columns, returns -1 if index out of bounds |
| `abstract  java.lang.String` | `getDisplayName()`             returns the name of the output format |
| `int` | `getDisplayRow(int index)`             returns the displayed index of the given row, depending on the order of rows, returns -1 if index out of bounds |
| `boolean` | `getEnumerateColNames()`             returns whether column names or numbers instead are enumerateed |
| `boolean` | `getEnumerateRowNames()`             returns whether row names or numbers instead are enumerateed |
| `java.lang.String` | `getHeader(java.lang.String key)`             returns the value associated with the given key, null if if cannot be found |
| `double` | `getMean(int col, int row)`             returns the mean at the given position, if the position is valid, otherwise 0 |
| `int` | `getMeanPrec()`             returns the current precision for the means |
| `int` | `getMeanWidth()`             returns the current width for the mean |
| `boolean` | `getPrintColNames()`             returns whether column names or numbers instead are printed |
| `boolean` | `getPrintRowNames()`             returns whether row names or numbers instead are printed |
| `boolean` | `getRemoveFilterName()`             returns whether the filter classname is removed from the dataset name |
| `int` | `getRowCount()`             returns the number of rows |
| `boolean` | `getRowHidden(int index)`             returns the hidden status of the row, if the index is valid, otherwise false |
| `java.lang.String` | `getRowName(int index)`             returns the name of the row, if the index is valid, otherwise null. |
| `int` | `getRowNameWidth()`             returns the current width for the row names |
| `int[]` | `getRowOrder()`             returns the current order of the rows, null means the default order |
| `boolean` | `getShowAverage()`             returns whether average per column is displayed or not |
| `boolean` | `getShowStdDev()`             returns whether std deviations are displayed or not |
| `int` | `getSignificance(int col, int row)`             returns the significance at the given position, if the position is valid, otherwise SIGNIFICANCE\_ATIE |
| `int` | `getSignificanceCount(int col, int type)`             counts the occurrences of the given significance type in the given column. |
| `int` | `getSignificanceWidth()`             returns the current width for the significance |
| `double` | `getStdDev(int col, int row)`             returns the std deviation at the given position, if the position is valid, otherwise 0 |
| `int` | `getStdDevPrec()`             returns the current standard deviation precision |
| `int` | `getStdDevWidth()`             returns the current width for the std dev |
| `int` | `getVisibleColCount()`             returns the number of visible columns |
| `int` | `getVisibleRowCount()`             returns the number of visible rows |
| `java.util.Enumeration` | `headerKeys()`             returns an enumeration of the header keys |
| `void` | `setColHidden(int index, boolean hidden)`             sets the hidden status of the column (if the index is valid) |
| `void` | `setColName(int index, java.lang.String name)`             sets the name of the column (if the index is valid) |
| `void` | `setColNameWidth(int width)`             sets the width for the column names (0 = optimal) |
| `void` | `setColOrder(int[] order)`             sets the ordering of the columns, null means default |
| `void` | `setCount(int index, double count)`             sets the count for the row (if the index is valid) |
| `void` | `setCountWidth(int width)`             sets the width for the counts (0 = optimal) |
| `void` | `setEnumerateColNames(boolean enumerate)`             sets whether the column names are prefixed with "(x)" where "x" is the index |
| `void` | `setEnumerateRowNames(boolean enumerate)`             sets whether to the row names or numbers instead are enumerateed |
| `void` | `setMean(int col, int row, double value)`             sets the mean at the given position (if the position is valid) |
| `void` | `setMeanPrec(int prec)`             sets the precision for the means |
| `void` | `setMeanWidth(int width)`             sets the width for the mean (0 = optimal) |
| `void` | `setPrintColNames(boolean print)`             sets whether the column names or numbers instead are printed. |
| `void` | `setPrintRowNames(boolean print)`             sets whether the row names or numbers instead are printed deactivating automatically sets m\_EnumerateColNames to TRUE. |
| `void` | `setRanking(int[][] wins)`             sets the ranking data based on the wins |
| `void` | `setRemoveFilterName(boolean remove)`             sets whether to remove the filter classname from the dataset name |
| `void` | `setRowHidden(int index, boolean hidden)`             sets the hidden status of the row (if the index is valid) |
| `void` | `setRowName(int index, java.lang.String name)`             sets the name of the row (if the index is valid) |
| `void` | `setRowNameWidth(int width)`             sets the width for the row names (0 = optimal) |
| `void` | `setRowOrder(int[] order)`             sets the ordering of the rows, null means default |
| `void` | `setShowAverage(boolean show)`             sets whether to display the average per column or not |
| `void` | `setShowStdDev(boolean show)`             sets whether to display the std deviations or not |
| `void` | `setSignificance(int col, int row, int value)`             sets the significance at the given position (if the position is valid) |
| `void` | `setSignificanceWidth(int width)`             sets the width for the significance (0 = optimal) |
| `void` | `setSize(int cols, int rows)`             clears the content of the matrix and sets the new size |
| `void` | `setStdDev(int col, int row, double value)`             sets the std deviation at the given position (if the position is valid) |
| `void` | `setStdDevPrec(int prec)`             sets the precision for the standard deviation |
| `void` | `setStdDevWidth(int width)`             sets the width for the std dev (0 = optimal) |
| `void` | `setSummary(int[][] nonSigWins, int[][] wins)`             sets the non-significant and significant wins of the resultsets |
| `java.lang.String` | `toString()`             returns the matrix as a string |
| `abstract  java.lang.String` | `toStringHeader()`             returns the header of the matrix as a string |
| `abstract  java.lang.String` | `toStringKey()`             returns returns a key for all the col names, for better readability if the names got cut off |
| `abstract  java.lang.String` | `toStringMatrix()`             returns the matrix as a string |
| `abstract  java.lang.String` | `toStringRanking()`             returns the ranking in a string representation |
| `abstract  java.lang.String` | `toStringSummary()`             returns the summary as string |

**Methods inherited from class java.lang.Object**

`equals, getClass, hashCode, notify, notifyAll, wait, wait, wait`

**Methods inherited from interface weka.core.RevisionHandler**

`getRevision`

**Field Detail**

### SIGNIFICANCE\_TIE

```
public static final int SIGNIFICANCE_TIE
```

:   tie

    **See Also:**: Constant Field Values

---


### SIGNIFICANCE\_WIN

```
public static final int SIGNIFICANCE_WIN
```

:   win

    **See Also:**: Constant Field Values

---


### SIGNIFICANCE\_LOSS

```
public static final int SIGNIFICANCE_LOSS
```

:   loss

    **See Also:**: Constant Field Values

---


### TIE\_STRING

```
public java.lang.String TIE_STRING
```

:   tie string

---


### WIN\_STRING

```
public java.lang.String WIN_STRING
```

:   win string

---


### LOSS\_STRING

```
public java.lang.String LOSS_STRING
```

:   loss string

---


### LEFT\_PARENTHESES

```
public java.lang.String LEFT_PARENTHESES
```

:   the left parentheses for enumerating cols/rows

---


### RIGHT\_PARENTHESES

```
public java.lang.String RIGHT_PARENTHESES
```

:   the right parentheses for enumerating cols/rows


**Constructor Detail**

### ResultMatrix

```
public ResultMatrix()
```

:   initializes the matrix as 1x1 matrix

---


### ResultMatrix

```
public ResultMatrix(int cols,
                    int rows)
```

:   initializes the matrix with the given dimensions

---


### ResultMatrix

```
public ResultMatrix(ResultMatrix matrix)
```

:   initializes the matrix with the values from the given matrix

    **Parameters:**: `matrix` - the matrix to get the values from


**Method Detail**

### getDisplayName

```
public abstract java.lang.String getDisplayName()
```

:   returns the name of the output format

---


### assign

```
public void assign(ResultMatrix matrix)
```

:   acquires the data from the given matrix

---


### clear

```
public void clear()
```

:   removes the stored data and the ordering, but retains the dimensions of
    the matrix

---


### setSize

```
public void setSize(int cols,
                    int rows)
```

:   clears the content of the matrix and sets the new size

    :   **Parameters:**: `cols` - the number of mean columns: `rows` - the number of mean rows

---


### setMeanPrec

```
public void setMeanPrec(int prec)
```

:   sets the precision for the means

---


### getMeanPrec

```
public int getMeanPrec()
```

:   returns the current precision for the means

---


### setStdDevPrec

```
public void setStdDevPrec(int prec)
```

:   sets the precision for the standard deviation

---


### getStdDevPrec

```
public int getStdDevPrec()
```

:   returns the current standard deviation precision

---


### setColNameWidth

```
public void setColNameWidth(int width)
```

:   sets the width for the column names (0 = optimal)

---


### getColNameWidth

```
public int getColNameWidth()
```

:   returns the current width for the column names

---


### setRowNameWidth

```
public void setRowNameWidth(int width)
```

:   sets the width for the row names (0 = optimal)

---


### getRowNameWidth

```
public int getRowNameWidth()
```

:   returns the current width for the row names

---


### setMeanWidth

```
public void setMeanWidth(int width)
```

:   sets the width for the mean (0 = optimal)

---


### getMeanWidth

```
public int getMeanWidth()
```

:   returns the current width for the mean

---


### setStdDevWidth

```
public void setStdDevWidth(int width)
```

:   sets the width for the std dev (0 = optimal)

---


### getStdDevWidth

```
public int getStdDevWidth()
```

:   returns the current width for the std dev

---


### setSignificanceWidth

```
public void setSignificanceWidth(int width)
```

:   sets the width for the significance (0 = optimal)

---


### getSignificanceWidth

```
public int getSignificanceWidth()
```

:   returns the current width for the significance

---


### setCountWidth

```
public void setCountWidth(int width)
```

:   sets the width for the counts (0 = optimal)

---


### getCountWidth

```
public int getCountWidth()
```

:   returns the current width for the counts

---


### setShowStdDev

```
public void setShowStdDev(boolean show)
```

:   sets whether to display the std deviations or not

---


### getShowStdDev

```
public boolean getShowStdDev()
```

:   returns whether std deviations are displayed or not

---


### setShowAverage

```
public void setShowAverage(boolean show)
```

:   sets whether to display the average per column or not

---


### getShowAverage

```
public boolean getShowAverage()
```

:   returns whether average per column is displayed or not

---


### setRemoveFilterName

```
public void setRemoveFilterName(boolean remove)
```

:   sets whether to remove the filter classname from the dataset name

---


### getRemoveFilterName

```
public boolean getRemoveFilterName()
```

:   returns whether the filter classname is removed from the dataset name

---


### setPrintColNames

```
public void setPrintColNames(boolean print)
```

:   sets whether the column names or numbers instead are printed.
    deactivating automatically sets m\_EnumerateColNames to TRUE.

    :   **See Also:**: `setEnumerateColNames(boolean)`

---


### getPrintColNames

```
public boolean getPrintColNames()
```

:   returns whether column names or numbers instead are printed

---


### setPrintRowNames

```
public void setPrintRowNames(boolean print)
```

:   sets whether the row names or numbers instead are printed
    deactivating automatically sets m\_EnumerateColNames to TRUE.

    :   **See Also:**: `setEnumerateRowNames(boolean)`

---


### getPrintRowNames

```
public boolean getPrintRowNames()
```

:   returns whether row names or numbers instead are printed

---


### setEnumerateColNames

```
public void setEnumerateColNames(boolean enumerate)
```

:   sets whether the column names are prefixed with "(x)" where "x" is
    the index

---


### getEnumerateColNames

```
public boolean getEnumerateColNames()
```

:   returns whether column names or numbers instead are enumerateed

---


### setEnumerateRowNames

```
public void setEnumerateRowNames(boolean enumerate)
```

:   sets whether to the row names or numbers instead are enumerateed

---


### getEnumerateRowNames

```
public boolean getEnumerateRowNames()
```

:   returns whether row names or numbers instead are enumerateed

---


### getColCount

```
public int getColCount()
```

:   returns the number of columns

---


### getVisibleColCount

```
public int getVisibleColCount()
```

:   returns the number of visible columns

---


### getRowCount

```
public int getRowCount()
```

:   returns the number of rows

---


### getVisibleRowCount

```
public int getVisibleRowCount()
```

:   returns the number of visible rows

---


### setColName

```
public void setColName(int index,
                       java.lang.String name)
```

:   sets the name of the column (if the index is valid)

    :   **Parameters:**: `index` - the index of the column: `name` - the name of the column

---


### getColName

```
public java.lang.String getColName(int index)
```

:   returns the name of the row, if the index is valid, otherwise null.
    if getPrintColNames() is FALSE then an empty string is returned or if
    getEnumerateColNames() is TRUE then the 1-based index surrounded by
    parentheses.

    :   **See Also:**: `setPrintColNames(boolean)`, `getPrintColNames()`, `setEnumerateColNames(boolean)`, `getEnumerateColNames()`

---


### setRowName

```
public void setRowName(int index,
                       java.lang.String name)
```

:   sets the name of the row (if the index is valid)

    :   **Parameters:**: `index` - the index of the row: `name` - the name of the row

---


### getRowName

```
public java.lang.String getRowName(int index)
```

:   returns the name of the row, if the index is valid, otherwise null.
    if getPrintRowNames() is FALSE then an empty string is returned or if
    getEnumerateRowNames() is TRUE then the 1-based index surrounded by
    parentheses.

    :   **See Also:**: `setPrintRowNames(boolean)`, `getPrintRowNames()`, `setEnumerateRowNames(boolean)`, `getEnumerateRowNames()`

---


### setColHidden

```
public void setColHidden(int index,
                         boolean hidden)
```

:   sets the hidden status of the column (if the index is valid)

    :   **Parameters:**: `index` - the index of the column: `hidden` - the hidden status of the column

---


### getColHidden

```
public boolean getColHidden(int index)
```

:   returns the hidden status of the column, if the index is valid, otherwise
    false

---


### setRowHidden

```
public void setRowHidden(int index,
                         boolean hidden)
```

:   sets the hidden status of the row (if the index is valid)

    :   **Parameters:**: `index` - the index of the row: `hidden` - the hidden status of the row

---


### getRowHidden

```
public boolean getRowHidden(int index)
```

:   returns the hidden status of the row, if the index is valid, otherwise
    false

---


### setCount

```
public void setCount(int index,
                     double count)
```

:   sets the count for the row (if the index is valid)

    :   **Parameters:**: `index` - the index of the row: `count` - the count for the row

---


### getCount

```
public double getCount(int index)
```

:   returns the count for the row. if the index is invalid then 0.

    :   **Parameters:**: `index` - the index of the row **Returns:**: the count for the row

---


### setMean

```
public void setMean(int col,
                    int row,
                    double value)
```

:   sets the mean at the given position (if the position is valid)

    :   **Parameters:**: `col` - the column of the mean: `row` - the row of the mean: `value` - the value of the mean

---


### getMean

```
public double getMean(int col,
                      int row)
```

:   returns the mean at the given position, if the position is valid,
    otherwise 0

---


### getAverage

```
public double getAverage(int col)
```

:   returns the average of the mean at the given position, if the position is
    valid, otherwise 0

---


### setStdDev

```
public void setStdDev(int col,
                      int row,
                      double value)
```

:   sets the std deviation at the given position (if the position is valid)

    :   **Parameters:**: `col` - the column of the std. deviation: `row` - the row of the std deviation: `value` - the value of the std deviation

---


### getStdDev

```
public double getStdDev(int col,
                        int row)
```

:   returns the std deviation at the given position, if the position is valid,
    otherwise 0

---


### setSignificance

```
public void setSignificance(int col,
                            int row,
                            int value)
```

:   sets the significance at the given position (if the position is valid)

    :   **Parameters:**: `col` - the column of the significance: `row` - the row of the significance: `value` - the value of the significance

---


### getSignificance

```
public int getSignificance(int col,
                           int row)
```

:   returns the significance at the given position, if the position is valid,
    otherwise SIGNIFICANCE\_ATIE

---


### getSignificanceCount

```
public int getSignificanceCount(int col,
                                int type)
```

:   counts the occurrences of the given significance type in the given
    column.

    :   **Parameters:**: `col` - the columnn to gather the information from: `type` - the significance type, WIN/TIE/LOSS

---


### setRowOrder

```
public void setRowOrder(int[] order)
```

:   sets the ordering of the rows, null means default

    :   **Parameters:**: `order` - the new order of the rows

---


### getRowOrder

```
public int[] getRowOrder()
```

:   returns the current order of the rows, null means the default order

    :   **Returns:**: the current order of the rows

---


### getDisplayRow

```
public int getDisplayRow(int index)
```

:   returns the displayed index of the given row, depending on the order of
    rows, returns -1 if index out of bounds

    :   **Parameters:**: `index` - the row to get the displayed index for **Returns:**: the real index of the row

---


### setColOrder

```
public void setColOrder(int[] order)
```

:   sets the ordering of the columns, null means default

    :   **Parameters:**: `order` - the new order of the columns

---


### getColOrder

```
public int[] getColOrder()
```

:   returns the current order of the columns, null means the default order

    :   **Returns:**: the current order of the columns

---


### getDisplayCol

```
public int getDisplayCol(int index)
```

:   returns the displayed index of the given col, depending on the order of
    columns, returns -1 if index out of bounds

    :   **Parameters:**: `index` - the column to get the displayed index for **Returns:**: the real index of the column

---


### toStringMatrix

```
public abstract java.lang.String toStringMatrix()
```

:   returns the matrix as a string

---


### toString

```
public java.lang.String toString()
```

:   returns the matrix as a string

    :   **Overrides:**: `toString` in class `java.lang.Object`
    :   **See Also:**: `toStringMatrix()`

---


### clearHeader

```
public void clearHeader()
```

:   removes all the header information

---


### addHeader

```
public void addHeader(java.lang.String key,
                      java.lang.String value)
```

:   adds the key-value pair to the header

    :   **Parameters:**: `key` - the name of the header value: `value` - the value of the header value

---


### getHeader

```
public java.lang.String getHeader(java.lang.String key)
```

:   returns the value associated with the given key, null if if cannot be
    found

    :   **Parameters:**: `key` - the key to retrieve the value for **Returns:**: the associated value

---


### headerKeys

```
public java.util.Enumeration headerKeys()
```

:   returns an enumeration of the header keys

    :   **Returns:**: all stored keys

---


### toStringHeader

```
public abstract java.lang.String toStringHeader()
```

:   returns the header of the matrix as a string

    :   **See Also:**: `m_HeaderKeys`, `m_HeaderValues`

---


### toStringKey

```
public abstract java.lang.String toStringKey()
```

:   returns returns a key for all the col names, for better readability if
    the names got cut off

---


### clearSummary

```
public void clearSummary()
```

:   clears the current summary data

---


### setSummary

```
public void setSummary(int[][] nonSigWins,
                       int[][] wins)
```

:   sets the non-significant and significant wins of the resultsets

    :   **Parameters:**: `nonSigWins` - the non-significant wins: `wins` - the significant wins

---


### toStringSummary

```
public abstract java.lang.String toStringSummary()
```

:   returns the summary as string

---


### clearRanking

```
public void clearRanking()
```

:   clears the currently stored ranking data

---


### setRanking

```
public void setRanking(int[][] wins)
```

:   sets the ranking data based on the wins

    :   **Parameters:**: `wins` - the wins

---


### toStringRanking

```
public abstract java.lang.String toStringRanking()
```

:   returns the ranking in a string representation


---


|  |  |  |
| --- | --- | --- |
| **Overview** \| **Package** \| **Class** \| **Tree** \| **Deprecated** \| **Index** \| **Help** \| **Weka's home** | |  |
| **PREV CLASS**   **NEXT CLASS** | **FRAMES**    **NO FRAMES**     **All Classes** |  |
| SUMMARY: NESTED \| FIELD \| CONSTR \| METHOD | DETAIL: FIELD \| CONSTR \| METHOD |  |


---
